# Contrasting Echocardiographic Trajectories with and Without Tafamidis in Wild-Type Transthyretin Amyloid Cardiomyopathy: A Real-World Observational Study with Two-Dimensional Speckle-Tracking Analysis

**DOI:** 10.3390/jcm15197441

**Published:** 2026-09-24

**Authors:** Inés Ramos González-Cristóbal, Irene Marco Clement, Ana Paola Espinoza, Patricia Landin Jimenez, Raquel Frias García-Lago, Lucía Galán Dávila, Marta Vigara García, María del Rosario Iguarán Bermúdez, Josebe Goirigolzarri Artaza, María Alejandra Restrepo Córdoba

**Affiliations:** 1 Cardiology Department, Hospital Clínico San Carlos, 28040 Madrid, Spain; irgonzalezcristobal@salud.madrid.org (I.R.G.-C.); irene.marco@salud.madrid.org (I.M.C.); espinozaanap@gmail.com (A.P.E.); patricialandin1303@gmail.com (P.L.J.); rfrias@salud.madrid.org (R.F.G.-L.); josebe.goirigolzarri@salud.madrid.org (J.G.A.); 2 Neurology Department, Hospital Clínico San Carlos, 28040 Madrid, Spain; lgalan@salud.madrid.org; 3 Geriatric Department, Hospital Clínico San Carlos, 28040 Madrid, Spain; marta.vigara@salud.madrid.org; 4 Internal Medicine Department, Hospital Clínico San Carlos, 28040 Madrid, Spain; mariarosario.iguaran@salud.madrid.org; 5 Instituto Cardiovascular, Hospital Clínico San Carlos, 28040 Madrid, Spain

**Keywords:** transthyretin amyloid cardiomyopathy, tafamidis, echocardiography, speckle-tracking, global longitudinal strain, left atrial strain

## Abstract

**Introduction and Objectives**: Transthyretin amyloid cardiomyopathy (ATTR-CM) is a progressive infiltrative disease with high morbidity and mortality in the absence of disease-specific therapy. We aimed to determine the impact of tafamidis on serial echocardiographic parameters—including two-dimensional speckle-tracking echocardiography (2D-STE) of the left ventricle (LV), right ventricle (RV) and left atrium (LA)—in patients with wild-type ATTR-CM followed in a real-world clinical setting, compared with a historical untreated cohort reflecting the natural disease course. **Patients and Methods**: Single-centre retrospective observational study comparing a historical, treatment-naïve cohort (n = 37) with a tafamidis-treated cohort (n = 22), for a total of 59 patients with confirmed ATTRwt-CM. Serial echocardiograms were performed at baseline and after a median follow-up of 22.4 months (untreated) and 18.2 months (treated). **Results**: Mean age was 87.0 (SD 4.8) years; 84.7% were male. Untreated patients were older (89.3 vs. 83.0 years; *p* < 0.001), had more advanced NAC staging (*p* = 0.016), and higher NT-proBNP (4291 vs. 1675 pg/mL; *p* = 0.011) and troponin I (*p* = 0.007). The untreated cohort showed significant worsening of posterior wall thickness (14.3 to 15.7 mm; *p* = 0.006), LV end-diastolic diameter (44.2 to 41.6 mm; *p* = 0.005), TAPSE (17.4 to 15.0 mm; *p* = 0.010), mitral E velocity (93.9 to 83.7 cm/s; *p* = 0.019), and LA contractile strain (8.5 to 6.4%; *p* = 0.014, Wilcoxon). All-cause mortality (70.3% vs. 9.1%; *p* < 0.001) and HF hospitalisation or IV diuretic requirement (78.4% vs. 4.5%; *p* < 0.001) were markedly higher in the untreated cohort. These parameters remained numerically stable in the tafamidis group (all *p* > 0.3). On direct between-group comparison of the magnitude of change, only LA contractile strain (Δ*p* = 0.045) and RV longitudinal strain (Δ*p* = 0.031) differed significantly between cohorts; LVEDD and LVEF approached significance (Δ*p* = 0.059 and 0.054), while the remaining parameters did not (Δ*p* > 0.18). **Conclusions**: Structural and functional echocardiographic parameters deteriorated significantly in the untreated cohort, while remaining numerically stable in tafamidis-treated patients. Serial 2D-STE incorporating RV and LA strain assessment provides a valuable tool for longitudinal monitoring in ATTRwt-CM.

## 1. Introduction

Transthyretin amyloid cardiomyopathy (ATTR-CM) is a progressive infiltrative cardiomyopathy resulting from deposition of misfolded transthyretin (TTR) fibrils within the myocardial interstitium, leading to biventricular wall thickening, diastolic dysfunction, conduction disease, and ultimately refractory heart failure [1,2]. The wild-type form (ATTRwt-CM) predominates in elderly patients and may account for up to 15% of heart failure with preserved ejection fraction in patients aged ≥ 80 years [3].

The landmark ATTR-ACT trial demonstrated that tafamidis—a TTR tetramer stabiliser—significantly reduced all-cause mortality, cardiovascular hospitalisations, and functional deterioration versus placebo in patients with ATTR-CM [4], leading to its regulatory approval. Despite this, data on the specific impact of tafamidis on serial echocardiographic parameters in real-world clinical practice remain limited, particularly using advanced imaging techniques such as two-dimensional speckle-tracking echocardiography (2D-STE).

Two-dimensional STE has emerged as a key tool for the diagnosis and monitoring of ATTR-CM [5,6]. Global longitudinal strain (GLS) of the LV, with its characteristic relative apical sparing pattern, is a sensitive marker of myocardial infiltration preceding LVEF deterioration [6]. More recently, RV longitudinal strain and LA strain parameters have been incorporated as complementary tools [7,8]. Rettl et al. demonstrated that tafamidis free acid 61 mg stabilised LV-GLS and LA reservoir strain versus a treatment-naïve cohort, with parallel clinical benefits [9]. Giblin et al. reported a significantly smaller absolute GLS deterioration in treated patients compared with controls [10]. Uemura et al. showed that baseline LA reservoir strain independently predicted cardiovascular events in tafamidis-treated ATTR-CM [8].

The present study aimed to assess the impact of tafamidis on serial echocardiographic parameters—including 2D-STE of the LV, RV, and LA—in a real-world cohort of ATTRwt-CM patients, compared with a historical untreated cohort reflecting the natural disease course. As a secondary objective, we analysed the impact of treatment on major clinical outcomes during follow-up.

## 2. Materials and Methods

### 2.1. Study Design and Population

Single-centre retrospective analysis of prospectively collected data from an IRB-approved registry, conducted over two years, from July 2023 to July 2025, at the Cardiac Amyloidosis Clinic at a tertiary hospital, reported in accordance with the STROBE guidelines [11]. We studied patients with confirmed ATTR-CM using the non-invasive algorithm by Gillmore et al., requiring Perugini grade ≥ 2 myocardial uptake on ^99^mTc-DPD scintigraphy in the absence of a monoclonal protein on serum and urine studies [12]. Patients with a confirmed pathogenic TTR variant on genetic testing and those with baseline echocardiographic data of insufficient quality for strain analysis were excluded.

Patients were classified into two groups: (I) historical treatment-naïve control cohort with patients followed in the clinic before disease-specific therapy was approved, (n = 37), and (II) tafamidis-treated cohort (n = 22), receiving tafamidis meglumine free acid 61 mg once daily. Of these, 3 patients had received tafamidis meglumine 20 mg QD until July 2023 as part of a Pfizer-supported compassionate use programme. The study was approved by the Institutional Ethics Committee, and all participants provided written informed consent.

### 2.2. Clinical Assessment and Staging

Baseline data collected included: age, sex, BMI, New York Heart Association (NYHA) class, comorbidities, and concomitant medications. Disease severity was staged using the National Amyloidosis Centre (NAC) staging system, based on N-terminal pro-B-type natriuretic peptide (NT-proBNP) and estimated glomerular filtration rate (eGFR) [13]. Laboratory parameters included serum creatinine, NT-proBNP, troponin, CA-125, and glycated haemoglobin.

### 2.3. Echocardiographic Protocol

Transthoracic echocardiograms were performed at baseline and at least 6 months of follow-up using high-end equipment (GE Vivid E9/E95) by certified operators following current ESC/EACVI guidelines [14,15]. The standard protocol was extended to include 2D-STE analysis of the LV, RV, and LA. All acquisitions were performed using the same equipment throughout the study period, and all 2D-STE analyses were conducted using the same version of GE EchoPAC dedicated software (GE Healthcare, Chicago, IL, USA). Frame rate settings were optimised for strain analysis in accordance with a standardised protocol applied consistently across both cohorts and the entire study period.

Right ventricular hypertrophy was defined as a free wall thickness > 5 mm and left ventricular hypertrophy as an interventricular septal or posterior wall thickness > 1.1 cm in men or >1.0 cm in women, in accordance with current EACVI/ASE recommendations for cardiac chamber quantification [14,15].

LV GLS was calculated as the mean of all LV segments across apical two-, three-, and four-chamber views [16]. RV longitudinal strain was measured in the free wall from the optimised apical four-chamber view [7]. LA strain components—reservoir, conduit, and contractile—were analysed exclusively in patients in sinus rhythm [7].

All analyses were performed blinded to baseline values and treatment group assignment.

### 2.4. Statistical Analysis

Continuous variables are expressed as mean (standard deviation, SD) or median (interquartile range, IQR) according to distribution. Categorical variables are presented as absolute frequencies and percentages. For comparisons between independent groups, Student’s *t*-test or Mann–Whitney U test was used. For intragroup longitudinal comparisons (baseline vs. follow-up), paired *t*-test or Wilcoxon signed-rank test were applied according to the Shapiro–Wilk test result for normality of differences. Between-group comparison of the magnitude of longitudinal change was performed using the same approach, applied to the per-patient change from baseline to follow-up (Δ = follow-up − baseline) for each parameter, with normality of Δ assessed via the Shapiro–Wilk test. Categorical variables were compared by Pearson chi-square test, Fisher’s exact test, or McNemar’s test as appropriate.

Univariable Cox proportional hazards regression was performed for both endpoints—all-cause mortality and HF hospitalisation or IV diuretic requirement. Full univariable results for both endpoints are provided in Supplementary Tables S1 and S2. Variables significant (*p* < 0.05) or showing a trend (*p* < 0.10) in the univariable analysis were considered candidates for multivariable modelling. A multivariable Cox model for HF hospitalisation or IV diuretic requirement was built including tafamidis treatment, age, and NAC stage, using a ridge penalisation (penalty = 0.1) to account for the limited number of events relative to covariates. Model discrimination was assessed using the concordance index (C-index).

All tests were two-sided; *p* < 0.05 was considered statistically significant and *p* < 0.10 was considered a statistical trend. Statistical analyses were performed using SPSS version 26.0 (IBM Corp., Armonk, NY, USA).

## 3. Results

### 3.1. Baseline Characteristics

Fifty-nine patients with ATTRwt-CM were included (Table 1). Mean age was 87.0 (SD 4.8) years, with marked male predominance (84.7%). Tafamidis-treated patients were significantly younger than untreated patients (83.0 vs. 89.3 years; *p* < 0.001). No significant differences were found in sex distribution (*p* = 0.134), NYHA class ≥ III (23.8% vs. 16.2%; *p* = 0.504), or the prevalence of major comorbidities between groups (Table 1).

Patients in the untreated cohort presented with more advanced disease at diagnosis: a higher proportion of NAC stage III (27.0% vs. 5.0%; *p* = 0.016), significantly higher NT-proBNP (4291 vs. 1675 pg/mL; *p* = 0.011), and higher troponin I (118 vs. 54 ng/L; *p* = 0.007). No significant differences in baseline echocardiographic parameters were observed between groups, except for a higher prevalence of RV hypertrophy in the tafamidis cohort (73.7% vs. 35.1%; *p* = 0.010). Baseline LA contractile strain tended to be lower in the tafamidis group (6.1 vs. 8.5%; *p* = 0.058). Median follow-up time was 22.4 months (IQR 15.8–32.3) in the untreated group and 18.2 months (IQR 13.3–32.7) in the treated group (*p* = 0.494).

### 3.2. Longitudinal Echocardiographic Changes—Within-Group Comparisons

In the untreated cohort, there was a significant increase in posterior wall thickness (14.3 to 15.7 mm; *p* = 0.006), along with a decrease in LV end-diastolic diameter (44.2 to 41.6 mm; *p* = 0.005). In contrast, neither parameter showed significant change in the tafamidis group (PW: 14.9 to 15.3 mm, *p* = 0.473; LVEDD: 42.6 to 41.9 mm, *p* = 0.924). IVS showed no significant change in either group. Regarding systolic function, LVEF remained stable in both cohorts. RV function assessed by TAPSE deteriorated significantly only in the untreated cohort (17.4 to 15.0 mm; *p* = 0.010), while it remained stable in the treated group (19.2 to 18.8 mm; *p* = 0.744). RV longitudinal strain showed a non-significant trend toward deterioration in the untreated cohort (15.3 to 13.1%; *p* = 0.182) and a non-significant trend toward improvement in the tafamidis group (15.3 to 18.8%; *p* = 0.094). LV GLS did not change significantly in either group (untreated: 12.7 to 11.8%, *p* = 0.325; treated: 11.4 to 11.2%, *p* = 0.886), though the magnitude of decline was numerically greater in the untreated cohort.

Regarding diastolic function, mitral E velocity declined significantly in the untreated cohort (93.9 to 83.7 cm/s; *p* = 0.019), but not in the treated group (90.5 to 89.3 cm/s; *p* = 0.719). The E/e’ ratio did not change significantly in either group.

In the LA strain analysis—restricted to patients in sinus rhythm—LA contractile strain (LASct) deteriorated significantly in the untreated cohort (8.5 to 6.4%; *p* = 0.014), while it remained stable in the tafamidis group (6.1 to 7.4%; *p* = 0.313). LA reservoir strain showed no significant change in either group. On direct between-group comparison of the magnitude of change for each parameter, only RV longitudinal strain and LA contractile strain differed significantly between cohorts (Table 2 and Figure 1).

### 3.3. Clinical Outcomes During Follow-Up

Clinical outcomes are summarised in Table 3. All-cause mortality was markedly higher in the untreated cohort (70.3% vs. 9.1%; *p* < 0.001), as was HF hospitalisation or IV diuretic requirement (78.4% vs. 4.5%; *p* < 0.001). No significant differences were observed in new-onset AF (16.2% vs. 31.8%; *p* = 0.194) or pacemaker implantation (27.0% vs. 15.0%; *p* = 0.346). Tafamidis treatment was the strongest independent predictor of both all-cause mortality and HF hospitalisation or IV diuretic requirement on univariable Cox regression, acting as a protective factor for both endpoints (mortality: HR 0.19, 95% CI 0.08–0.47; *p* < 0.001; HF hospitalisation: HR 0.13, 95% CI 0.05–0.33; *p* < 0.001). Age, NAC stage, and log (NT-proBNP) were additionally significant for both endpoints, while creatinine and baseline TAPSE reached significance only for mortality. Full univariable results are provided in Supplementary Tables S1 and S2. On multivariable analysis for HF hospitalisation or IV diuretic requirement, adjusted for age and NAC stage (n = 56, events = 30), tafamidis maintained its independent protective effect (HR 0.16, 95% CI 0.06–0.44; *p* < 0.001; C-index 0.878) (Table 4).

## 4. Discussion

This real-world observational study describes the echocardiographic and clinical evolution of two ATTRwt-CM cohorts, with and without tafamidis. Within the untreated cohort, posterior wall thickness, LVEDD, TAPSE, mitral E velocity and LA contractile strain all deteriorated significantly over follow-up, consistent with the expected natural history of the disease. Within the tafamidis cohort, none of these parameters changed significantly, and RV longitudinal strain showed a non-significant trend toward improvement. Critically, the two cohorts differed substantially at baseline in age, NAC stage, NT-proBNP, troponin, and RV hypertrophy prevalence—all established markers of disease severity and prognosis in this condition. These imbalances reflect the systematic difference in diagnostic stage at which the two groups were identified: treated patients were younger and diagnosed earlier, at a time when structured ATTRwt-CM diagnostic programmes were more widely established. In this context, preservation of a parameter in the treated cohort and deterioration in the untreated cohort cannot itself constitute evidence of a pharmacological effect. The more appropriate approach is the direct between-group comparison of the magnitude of longitudinal change (Δ), which controls for baseline differences by design. Only RV longitudinal strain (Δ*p* = 0.031) and LA contractile strain (Δ*p* = 0.045) showed significantly divergent trajectories; LVEDD and LVEF approached significance (Δ*p* = 0.059 and 0.054, respectively). These between-group differences likely reflect a combination of biological signal and residual confounding, and should be interpreted as hypothesis-generating rather than confirmatory.

From an echocardiographic standpoint, our structural findings are consistent with those reported by Restelli et al. [17], who demonstrated stable LV mass and echocardiographic parameters over a two-year period in tafamidis-treated ATTR-CM patients using mixed-effects models. Similarly, Rettl et al. [9] reported stabilisation of IVS thickness and GLS in both tafamidis formulations versus a treatment-naïve cohort where significant progression was observed (LV-GLS: 11.71 vs. 10.59%; *p* = 0.001; IVS: 18.8 vs. 19.9 mm; *p* < 0.001). The between-group comparison for LV-GLS in their cohort reached statistical significance (*p* = 0.030 for tafamidis free acid 61 mg), a finding we could not replicate, likely due to our smaller sample size (n = 21–33 pairs for GLS analyses). On direct between-group comparison, the magnitude of GLS change did not differ significantly between cohorts either (Δ*p* = 0.575).

The absence of a significant between-group difference in LV-GLS (Δ*p* = 0.575) warrants specific comment, given that this is the most extensively validated 2D-STE index in ATTR-CM. Several non-mutually exclusive explanations may account for this finding. First, limited statistical power: the number of analysable paired studies for LV GLS was n = 21 (untreated) and n = 20 (treated), substantially constraining power to detect a between-group difference in the magnitude previously reported (~1.1% difference in GLS change [9]). Second, restricted dynamic range: both cohorts presented with already impaired baseline LV GLS values (12.7% and 11.4%, respectively) for confirmed ATTRwt-CM, limiting room for further measurable deterioration over the follow-up period. Third, chamber-specific disease progression: RV longitudinal strain and LA contractile strain, which showed significant between-group differences (Δ*p* = 0.031 and 0.045), may reflect a pattern of right-sided and atrial amyloid infiltration that is particularly active within the timeframe of this study. This observation warrants prospective investigation in adequately powered studies.

RV involvement in ATTR-CM is increasingly recognised as a determinant of prognosis [18], and RV amyloid infiltration may progress independently of LV remodelling. In our cohort, we observed a significant decline in the TAPSE in the untreated group (17.4 to 15.0 mm; *p* = 0.010), with preservation in treated patients (19.2 to 18.8 mm; *p* = 0.744). Furthermore, there was a trend toward improvement in RV longitudinal strain in the tafamidis group (15.3 to 18.8%; *p* = 0.094). These findings—supported by a significant between-group difference in the magnitude of change (Δ*p* = 0.031)—suggest a potential benefit of tafamidis on RV function that warrants prospective investigation in larger cohorts.

A novel finding of our study is the significant deterioration of LA contractile strain in the untreated cohort, with preservation in the tafamidis group. Given the high prevalence of AF in our cohort (65.5% overall), this analysis was necessarily restricted to sinus rhythm patients, which limits statistical power. However, the direction of the finding is biologically plausible: LA amyloid infiltration impairs both the reservoir and the contractile components of LA function [8,19], and stabilisation of amyloid infiltration by tafamidis would be expected to preserve LA mechanical function. Uemura et al. [8] demonstrated that baseline LA reservoir strain independently predicts cardiovascular events in tafamidis-treated ATTR-CM, reinforcing the clinical relevance of LA strain assessment. Rettl et al. [9] reported stabilisation of LASr in both tafamidis formulations versus deterioration in the naïve cohort. Our data extend these findings by showing that the contractile component may be particularly sensitive to detecting echocardiographic progression in this population. This was supported by direct between-group comparison of the magnitude of change, which was significant for the contractile component (Δ*p* = 0.045) but not for the reservoir component (Δ*p* = 0.312).

The large differences in clinical outcomes between groups must be interpreted cautiously. Because inclusion in the echocardiographic follow-up analysis required survival to at least 6 months, a survival bias favouring healthier patients cannot be entirely excluded, particularly in the untreated cohort given its high mortality; however, mortality was similar between untreated patients with and without a follow-up echocardiogram (70% vs. 75%), and most deaths among those with paired studies occurred well after the follow-up echo (median 234 days), arguing against a strong survival bias. The untreated cohort was significantly older (89.3 vs. 83.0 years), with more advanced NAC staging and higher biomarker levels, consistent with the real-world reality that tafamidis access has been predominantly limited to earlier-diagnosed patients. These baseline imbalances are inherent to the retrospective non-randomised design and preclude any causal attribution of the outcome differences. The multivariable survival analysis reinforces the main message of this study. After adjustment for age and NAC stage—the two main baseline imbalances between cohorts—tafamidis retained a strong and statistically significant independent protective effect against HF hospitalisation or IV diuretic requirement (HR 0.16; *p* < 0.001), with excellent overall model discrimination (C-index 0.878). These findings are consistent with the benefit demonstrated in the ATTR-ACT trial [4] and with subsequent real-world series showing preserved tafamidis efficacy across disease stages [20] and suggest that the observed differences in clinical outcomes are not entirely explained by baseline imbalances between cohorts.

Nevertheless, the baseline imbalances between cohorts—particularly in age, NAC stage, and biomarker levels—substantially limit the conclusions that can be drawn from the clinical outcome data, which should be regarded as contextual rather than confirmatory.

### Limitations

The main limitations of this study are as follows. (I) The single-centre retrospective design with limited sample size constrains statistical power to detect differences in continuous variables. The small number of analysable pairs per strain parameter should be considered when interpreting the strain results. (II) Baseline imbalances in age, NAC stage, NT-proBNP, troponin, and RV hypertrophy prevalence are inherent to the historical control design and represent a source of residual confounding that cannot be fully adjusted for statistically; formal causal inference therefore cannot be established. (III) Formal intra- and interobserver reproducibility analyses for LV GLS, RV longitudinal strain, and LA strain were not conducted in this study [21,22]. (IV) Extrinsic mechanical factors must also be acknowledged. In this elderly, male-predominant cohort, anterior chest wall morphology may influence cardiac geometry and myocardial deformation measurements independently of intrinsic myocardial disease [23]. (V) LA strain analysis was restricted to patients in sinus rhythm, given that >65% of the cohort had baseline AF. (VI) Between-group comparisons of the magnitude of change were performed across 13 echocardiographic parameters without correction for multiple testing; the two nominally significant differences identified (RV longitudinal strain and LA contractile strain) should be regarded as hypothesis-generating rather than confirmatory. (VII) Inclusion in the echocardiographic follow-up analysis required a minimum 6-month interval; mortality was similar between untreated patients with and without paired studies (70% vs. 75%), arguing against substantial survival bias.

## 5. Conclusions

In the historical cohort, echocardiographic parameters deteriorated significantly and clinical outcomes were considerably worse, consistent with the progressive natural history of ATTRwt-CM. In tafamidis-treated patients, echocardiographic decline was substantially more attenuated, with numerically stable values across most parameters and significantly divergent trajectories for RV longitudinal strain and LA contractile strain on direct between-group comparison. Given the marked baseline imbalances inherent to the historical-control design, these observations are best interpreted as supportive of a potential stabilising effect of tafamidis on cardiac mechanics, while acknowledging that causal attribution is not possible in a retrospective non-randomised study. Serial 2D-STE with assessment of RV function and LA strain provides a valuable tool for longitudinal monitoring in ATTRwt-CM, and the divergent trajectories of RV and LA contractile strain identified here represent candidates for prospective validation as markers of disease progression in this population.

## Figures and Tables

**Figure 1:**
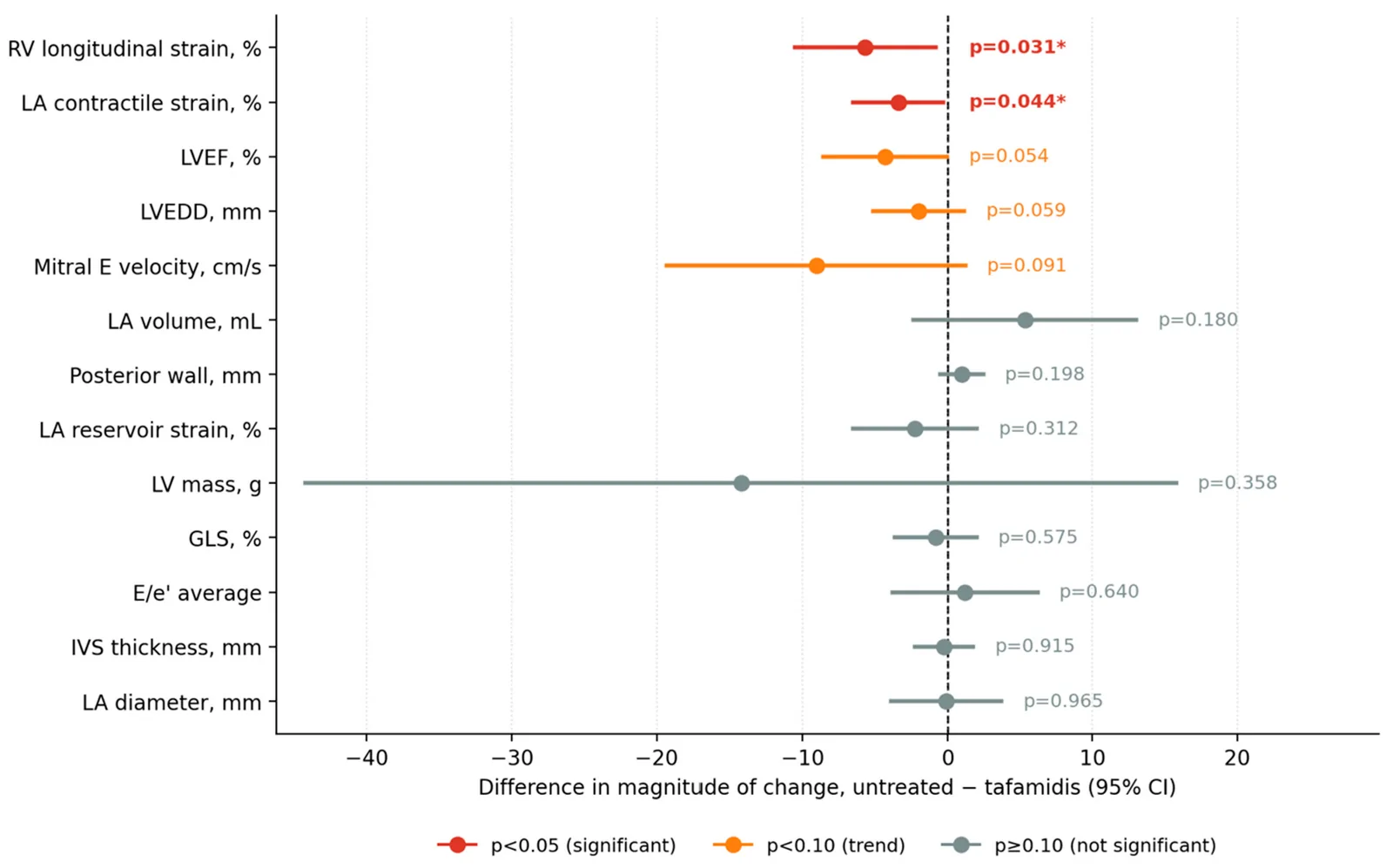
Between-group comparison of the magnitude of longitudinal echocardiographic change. Points represent the mean difference in Δ between the untreated and tafamidis-treated cohorts; horizontal lines indicate the 95% confidence interval, and the dashed vertical line indicates no difference. Red: *p* < 0.05; orange: *p* < 0.10; grey: *p* ≥ 0.10. GLS, global longitudinal strain; LA, left atrium; LVEDD, LV end-diastolic diameter; LVEF, LV ejection fraction; RV, right ventricle. * *p* < 0.05.

**Table 1:** Baseline characteristics by treatment group.

Variable	Total (n = 59)	No Tafamidis (n = 37)	Tafamidis (n = 22)	*p*
**Clinical Parameters**				
Age, years, mean (SD)	87.0 (4.8)	89.3 (3.8)	83.0 (3.4)	<0.001
Male sex, n (%)	50 (84.7)	29 (78.4)	21 (95.5)	0.134
NYHA class ≥ III, n (%)	11 (19.3)	6 (16.2)	5 (23.8)	0.504
NAC stage I/II/III, n (%)	21/25/11	9/18/10	12/7/1	0.016
Hypertension, n (%)	39 (67.2)	27 (73.0)	12 (54.5)	0.254
Diabetes mellitus, n (%)	13 (22.4)	9 (24.3)	4 (19.0)	0.751
Dyslipidaemia, n (%)	33 (56.9)	20 (54.1)	13 (61.9)	0.594
Coronary artery disease, n (%)	13 (22.8)	7 (18.9)	6 (28.6)	0.515
Peripheral neuropathy, n (%)	15 (26.3)	7 (18.9)	8 (38.1)	0.129
Carpal tunnel syndrome, n (%)	23 (39.7)	12 (32.4)	11 (52.4)	0.168
Baseline AF/flutter, n (%)	38 (65.5)	25 (67.6)	13 (61.9)	0.776
**Laboratory Values**				
Creatinine, mg/dL, mean (SD)	1.29 (0.45)	1.33 (0.52)	1.24 (0.31)	0.474
NT-proBNP, pg/mL, median (IQR)	3620 (1564–6618)	4291 (2366–8878)	1675 (1217–3608)	0.011
Troponin I, ng/L, median (IQR)	80 (47–118)	118 (88–209)	54 (41–69)	0.007
**Baseline Echocardiography**				
LVEDD, mm, mean (SD)	43.9 (6.3)	44.5 (5.7)	42.8 (7.2)	0.313
IVS thickness, mm, mean (SD)	16.5 (3.9)	16.0 (4.1)	17.4 (3.6)	0.187
Posterior wall, mm, mean (SD)	14.1 (3.5)	13.8 (3.5)	14.7 (3.6)	0.376
LVEF, %, mean (SD)	54.3 (10.4)	53.0 (11.5)	56.2 (8.5)	0.253
TAPSE, mm, mean (SD)	18.0 (4.9)	17.5 (4.9)	18.8 (4.9)	0.374
LA volume, mL, mean (SD)	51.0 (17.4)	53.7 (19.7)	46.2 (12.5)	0.118
GLS, %, mean (SD)	11.8 (3.8)	11.9 (3.5)	11.8 (4.1)	0.959
LA reservoir strain, %, mean (SD)	9.9 (5.5)	10.2 (5.7)	9.4 (5.2)	0.576
LA contractile strain, %, mean (SD)	7.5 (4.6)	8.5 (4.9)	6.1 (3.5)	0.058
RV longitudinal strain, %, mean (SD)	14.1 (5.0)	13.6 (4.7)	14.8 (5.4)	0.449
RV hypertrophy, n (%)	27 (48.2)	13 (35.1)	14 (73.7)	0.010
Follow-up, months, median (IQR)	—	22.4 (15.8–32.3)	18.2 (13.3–32.7)	0.494

AF, atrial fibrillation/flutter; CAD, coronary artery disease; CTS, carpal tunnel syndrome; GLS, global longitudinal strain; IQR, interquartile range; IVS, interventricular septum; LA, left atrium; LVEDD, LV end-diastolic diameter; LVEF, LV ejection fraction; NYHA, New York Heart Association; RV, right ventricle; SD, standard deviation; TAPSE, tricuspid annular plane systolic excursion.

**Table 2:** Serial echocardiographic parameters at baseline and follow-up.

Variable	No Tafamidis			Tafamidis			
	Baseline	Follow-Up	*p*	Baseline	Follow-Up	*p*	Δ*p*
LVEDD, mm (n = 35/n = 21)	44.2 (5.8)	41.6 (6.2)	0.005 *	42.6 (7.3)	41.9 (5.5)	0.924	0.059
IVS thickness, mm (n = 35/n = 21)	16.5 (4.0)	16.9 (3.8)	0.251	17.7 (3.4)	18.3 (3.4)	0.409	0.915
Posterior wall, mm (n = 35/n = 21)	14.3 (3.4)	15.7 (4.5)	0.006 *	14.9 (3.5)	15.3 (3.0)	0.473	0.198
LV mass, g (n = 26/n = 18)	168.4 (67.5)	170.0 (56.8)	0.855	157.4 (50.8)	173.3 (49.5)	0.222	0.358
LVEF, % (n = 35/n = 21)	54.6 (11.7)	51.7 (12.4)	0.093	56.4 (8.7)	57.8 (8.7)	0.326	0.054
GLS, % (n = 21/n = 20)	12.7 (3.7)	11.8 (3.7)	0.325	11.4 (4.2)	11.2 (3.4)	0.886	0.575
TAPSE, mm (n = 25/n = 18)	17.4 (4.7)	15.0 (4.6)	0.010 *	19.2 (5.1)	18.8 (4.3)	0.744	0.055
RV longitudinal strain, % (n = 18/n = 17)	15.3 (4.1)	13.1 (6.1)	0.182	15.3 (5.7)	18.8 (6.1)	0.094	0.031 *
Mitral E velocity, cm/s (n = 32/n = 19)	93.9 (26.7)	83.7 (20.8)	0.019 *	90.5 (26.8)	89.3 (29.0)	0.719	0.091
E/e’ average (n = 29/n = 17)	18.3 (7.3)	19.0 (7.1)	0.741	18.4 (7.2)	17.9 (5.3)	0.734	0.640
LA diameter, mm (n = 30/n = 20)	46.3 (5.4)	47.6 (8.6)	0.298	43.4 (6.0)	44.7 (4.5)	0.426	0.965
LA volume, mL (n = 30/n = 20)	54.7 (20.5)	57.6 (21.6)	0.227	46.2 (12.8)	43.7 (14.8)	0.442	0.180
LA reservoir strain, % (n = 30/n = 21)	10.4 (6.0)	9.4 (5.9)	0.328	9.7 (5.1)	10.9 (7.1)	0.536	0.312
LA contractile strain, % (n = 30/n = 21)	8.5 (5.1)	6.4 (3.8)	0.014 *	6.1 (3.6)	7.4 (3.7)	0.313	0.045 *

Data are expressed as mean (SD). Baseline values in Table 2 may differ from Table 1, as Table 2 includes only patients with a paired baseline and follow-up measurement for each variable; Δ*p*: *p*-value for the between-group comparison of the magnitude of longitudinal change (independent-samples *t*-test or Mann–Whitney U test on the per-patient change from baseline, according to distribution). GLS, global longitudinal strain; IVS, interventricular septum; LA, left atrium; LAS, left atrial strain; LVEDD, LV end-diastolic diameter; LVEF, LV ejection fraction; PW, posterior wall; RV, right ventricle; SD, standard deviation; TAPSE, tricuspid annular plane systolic excursion. * *p* < 0.05.

**Table 3:** Clinical outcomes during follow-up by treatment group.

Outcome	No Tafamidis (n = 37)	Tafamidis (n = 22)	*p*
All-cause mortality, n (%)	26 (70.3)	2 (9.1)	<0.001
HF hospitalisation or IV diuretic, n (%)	29 (78.4)	1 (4.5)	<0.001
New-onset atrial fibrillation, n (%)	6 (16.2)	7 (31.8)	0.194
Pacemaker implantation, n (%)	10 (27.0)	3 (15.0)	0.346

AF, atrial fibrillation; HF, heart failure; IV, intravenous.

**Table 4:** Multivariable Cox proportional hazards regression—HF hospitalisation or IV diuretic requirement (n = 56, events = 30, C-index = 0.878).

Variable	HR (95% CI)	*p*
Tafamidis (vs untreated)	0.16 (0.06–0.44)	<0.001
Age (per year)	1.00 (0.92–1.08)	0.974
NAC stage (per unit)	1.47 (0.92–2.34)	0.105

C-index: concordance index. HR, hazard ratio; CI, 95% confidence interval. Log-rank *p* < 0.001 for tafamidis vs. untreated (Kaplan–Meier).

## Data Availability

Data are not publicly available due to patient privacy restrictions but are available from the corresponding author upon reasonable request.

## Supplementary Materials

The following supporting information can be downloaded at: https://www.mdpi.com/article/10.3390/jcm15197441/s1, Table S1. Univariable Cox proportional hazards regression—All-cause mortality. Table S2. Univariable Cox proportional hazards regression—HF hospitalisation or IV diuretic requirement.
