# Supplementary material for: Contrasting Echocardiographic Trajectories with and Without Tafamidis in Wild-Type Transthyretin Amyloid Cardiomyopathy: A Real-World Observational Study with Two-Dimensional Speckle-Tracking Analysis
Source: J Clin Med. 2026 Sep 24;15(19):7441. doi: 10.3390/jcm15197441 (PMC13666391; doi:10.3390/jcm15197441)
Supplement: Supplementary file 1 [file jcm-15-07441-s001.zip › jcm-4517964-supplementary.pdf]

Supplementary Material

Table S1. Univariable Cox proportional hazards regression — All-cause mortality.

Variable	HR (95% CI)	p
Tafamidis (vs untreated)	0.19 (0.08–0.47)	<0.001
Age (per year)	1.14 (1.06–1.23)	<0.001
Male sex	1.50 (0.54–4.17)	0.433
NYHA class ≥III	1.19 (0.52–2.74)	0.679
Gilmore stage (per unit)	1.97 (1.25–3.13)	0.004
log(NT-proBNP)	1.62 (1.18–2.22)	0.003
Creatinine (per mg/dL)	1.96 (1.02–3.75)	0.043
Troponin I (per ng/L)	1.00 (1.00–1.00)	0.089
LVEF (per %)	0.98 (0.94–1.01)	0.146
IVS thickness (per mm)	1.03 (0.95–1.12)	0.469
TAPSE (per mm)	0.92 (0.86–1.00)	0.039
GLS (per %)	0.97 (0.88–1.07)	0.551
LA reservoir strain (per %)	1.01 (0.95–1.07)	0.711
LA contractile strain (per %)	1.07 (1.00–1.14)	0.068
RV hypertrophy	0.78 (0.40–1.54)	0.480
Hypertension	1.24 (0.59–2.58)	0.567

HR, hazard ratio; CI, confidence interval. log(NT-proBNP): natural logarithm of NT-proBNP.

Table S2. Univariable Cox proportional hazards regression — HF hospitalisation or IV diuretic requirement.

Variable	HR (95% CI)	p
Tafamidis (vs untreated)	0.13 (0.05–0.33)	<0.001
Age (per year)	1.10 (1.03–1.18)	0.007
Male sex	0.53 (0.23–1.19)	0.121
NYHA class ≥III	1.22 (0.53–2.80)	0.644
Gilmore stage (per unit)	2.01 (1.27–3.18)	0.003
log(NT-proBNP)	1.56 (1.12–2.17)	0.009
Creatinine (per mg/dL)	1.35 (0.66–2.76)	0.408
Troponin I (per ng/L)	1.00 (1.00–1.00)	0.087
LVEF (per %)	0.99 (0.95–1.02)	0.375
IVS thickness (per mm)	0.95 (0.87–1.03)	0.199
TAPSE (per mm)	0.95 (0.88–1.01)	0.108
GLS (per %)	1.02 (0.93–1.11)	0.727
LA reservoir strain (per %)	0.99 (0.93–1.06)	0.778
LA contractile strain (per %)	1.05 (0.98–1.12)	0.153
RV hypertrophy	0.79 (0.41–1.53)	0.486
Hypertension	1.73 (0.82–3.63)	0.148

HR, hazard ratio; CI, confidence interval. log(NT-proBNP): natural logarithm of NT-proBNP. Troponin I available in 23/59 patients.
